# Gut–microbiota–microglia–brain interactions in Alzheimer’s disease: knowledge-based, multi-dimensional characterization

**DOI:** 10.1186/s13195-021-00917-1

**Published:** 2021-10-20

**Authors:** QuanQiu Wang, Pamela B. Davis, Xin Qi, Shu G. Chen, Mark E. Gurney, George Perry, P. Murali Doraiswamy, Rong Xu

**Affiliations:** 1 Center for Artificial Intelligence in Drug Discovery, School of Medicine, Case Western Reserve University, 2103 Cornell Rd, Cleveland, OH 44106 USA; 2 Center for Community Health Integration, Division of General Medical Sciences, School of Medicine, Case Western Reserve University, Cleveland, OH USA; 3 Department of Physiology and Biophysics, School of Medicine, Case Western Reserve University, Cleveland, OH USA; 4 Department of Pathology, School of Medicine, Case Western Reserve University, Cleveland, OH USA; 5 Tetra Therapeutics, Grand Rapids, MI USA; 6 College of Sciences, The University of Texas at San Antonio, San Antonio, TX USA; 7 Duke University School of Medicine and the Duke Institute for Brain Sciences, Durham, NC 27710 USA

**Keywords:** Gut microbiota, Neurodegeneration, Microglia, Microbial metabolites, Systems biology, Networks

## Abstract

**Background:**

Interactions between the gut microbiota, microglia, and aging may modulate Alzheimer’s disease (AD) pathogenesis but the precise nature of such interactions is not known.

**Methods:**

We developed an integrated multi-dimensional, knowledge-driven, systems approach to identify interactions among microbial metabolites, microglia, and AD. Publicly available datasets were repurposed to create a multi-dimensional knowledge-driven pipeline consisting of an integrated network of microbial metabolite–gene–pathway–phenotype (MGPPN) consisting of 34,509 nodes (216 microbial metabolites, 22,982 genes, 1329 pathways, 9982 mouse phenotypes) and 1,032,942 edges.

**Results:**

We evaluated the network-based ranking algorithm by showing that abnormal microglia function and physiology are significantly associated with AD pathology at both genetic and phenotypic levels: AD risk genes were ranked at the top 6.4% among 22,982 genes, *P* < 0.001. AD phenotypes were ranked at the top 11.5% among 9982 phenotypes, *P* < 0.001. A total of 8094 microglia–microbial metabolite–gene–pathway–phenotype–AD interactions were identified for top-ranked AD-associated microbial metabolites. Short-chain fatty acids (SCFAs) were ranked at the top among prioritized AD-associated microbial metabolites. Through data-driven analyses, we provided evidence that SCFAs are involved in microglia-mediated gut–microbiota–brain interactions in AD at both genetic, functional, and phenotypic levels.

**Conclusion:**

Our analysis produces a novel framework to offer insights into the mechanistic links between gut microbial metabolites, microglia, and AD, with the overall goal to facilitate disease mechanism understanding, therapeutic target identification, and designing confirmatory experimental studies.

**Supplementary Information:**

The online version contains supplementary material available at 10.1186/s13195-021-00917-1.

## Introduction

Alzheimer’s disease (AD) is the leading cause of dementia and the most common neurodegenerative disorder, affecting over 5.5 million people in the USA and 47 million people worldwide [1]. AD is complex, with genetic, epigenetic, and environmental factors contributing to disease susceptibility and progression [2].

Trillions of bacteria in the human body (human microbiota) may affect human health and diseases by modulating host functions through small molecule metabolites. Undigested dietary components are fermented by microbiota to produce a wide array of metabolites such as bile acids, choline, and short-chain fatty acids (SCFAs) that are essential for human health [3–5]. Metabolite activities of gut microbiota provide a mechanistic connection between environmental factors and brain function and behavior [6–8]. The gut microbiota of AD patients has altered microbial diversity and is compositionally distinct from control age- and sex-matched individuals [9–11]. Recent studies showed that altered serum levels of bile acids, lipopolysaccharide, SCFAs, and trimethylamine-N-oxide (TMAO) were associated with cognitive impairment in AD [12–15].

In AD, microglia are involved in amyloid-β (Aβ) clearance in the brain and many innate immunity genes are associated with the risk of sporadic AD [16]. Microglia are the main neuroimmune cells involved in the development, normal functioning, aging, and injury of the central nervous system [16–18]. Gene variants in TREM2 and CD33 that modulate macrophage and microglial function increase the risk for late-onset AD [19]. There is increasing evidence that the interactions between the gut microbiota and brain innate immune system (gut–immune–brain axis) may modulate AD pathogenesis through microglial maturation and function [20–22]. Short-chain fatty acids (SCFAs), the end products of fermentation of dietary fibers by gut microbiota, play a major role in the maintenance of gut and immune homeostasis [23, 24]. SCFAs may play a key role in microbiota–gut–brain crosstalk. In vitro administration of SCFAs (microbial fermentation metabolites) regulated microglia homeostasis and obstructed Aβ protein aggregation [25]. Supplementation of SCFAs in germ-free mice rescued the immature genetic and morphological phenotype of microglia [20]. Despite such growing links, the mechanisms underlying how gut microbial metabolites including SCFAs interact with microglia and host genetics in promoting or protecting against AD remain largely unknown.

We have previously demonstrated that data-driven computational systems approaches have the potential in uncovering mechanistic links between microbial metabolites and human diseases [26–29]. For example, in a prior study, we provided evidence that trimethylamine N-oxide (TMAO), a human gut microbial metabolite of dietary meat and fat, was linked to AD [27], a finding that was subsequently confirmed by an experimental study [15]. In this study, we significantly expanded our prior work to produce the first comprehensive, multi-dimensional, systems framework of analyzing and identifying complex interrelationships among gut microbial metabolites, microglia, and AD at both genetic and functional levels. We first constructed a multi-model context-sensitive network to model complex and heterogeneous interrelationships among microbial metabolites, genes, pathways, and disease phenotypes. Then network-based prioritization algorithm prioritized microbial metabolites based on their relevance to microglia physiology and functions in AD. The overall goal of this study was to identify potential microglia–microbial metabolite–gene–pathway–phenotype interactions in AD with supporting evidence at genetic, functional, and phenotypic levels, which can set a foundation for others to conduct hypothesis-driven studies to test these interactions in experimental models or clinical samples.

## Methods

### Overview

**Fig. 1:**
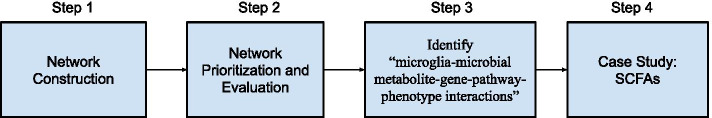
The integrated metabolite–gene–pathway–phenotype network (MGPPN) with labeled data resources. The goal of this study is to understand how microbial metabolites affect AD-specific phenotype through interactions among microglia, host genetics, and genetic pathways

The overall experiment (Fig. 1) consisted of (1) *network construction*: an integrated network of metabolite–gene–pathway–phenotype (MGPPN) was constructed from multiple data resources to model and capture the complex and heterogeneous interrelationships among microbial metabolites, genes, pathways, and disease phenotypes; (2) *network-based prioritization*: microbial metabolites were prioritized from MGPPN based on their relevance to microglia physiology and functions. The algorithm was evaluated using known AD-specific phenotypes, genes, and microbial metabolites. (3) *Identification of microglia–microbial metabolite–gene–pathway–phenotype interactions in AD*: Based on the top-ranked microglia-associated microbial metabolites, we identified interactions among microglia, microbial metabolites, genes, pathways, and phenotypes (“microglia-microbial metabolite-gene-pathway-phenotype interactions”) in AD. (4) *Case study of SCFAs*: We analyzed how SCFAs are involved in the microglia–microbiota–brain interactions in AD.

#### Network construction

MGPPN consisted of four sub-networks: human metabolite–gene network (MGN), mouse phenotype–gene network (PhenGN), protein–protein interaction network (PPIN), and pathway–gene network (PathGN). The numbers of nodes (metabolites, genes, pathways, phenotypes) and edges (connections between nodes) of each sub-network are shown in Table 1.

**Table 1:** Numbers of nodes and edges on each sub-network of MPGGN

Sub-network of MGPPN	Nodes	Edges
Human metabolite–gene network (MGN)	216 metabolites, 11,914 genes	67,012
Mouse phenotype–gene network (PhenGN)	9982 phenotypes, 11,021 genes	517,381
Protein–protein interaction network (PPIN)	22,982 genes	382,256
Pathway–gene network (PathGN)	8868 genes, 1329 pathways	66,293

##### Human microbial metabolite–gene network (MGN)

Metabolites found in the human body can originate from different sources, including human hosts, plants, foods, microbes, toxins, pollutants, cosmetics, and medications, among others. In this study, we focused on microbial metabolites that are present in the human body and that are produced (not necessarily exclusively) by microbes. Human Metabolome Database (HMDB) contains 114,304 small molecule metabolites found in the human body, among which 220 metabolites were originated in microbiota [30] (data accessed in October 2020). Based on manual curation effort in HMDB, 12 microbial metabolites are associated with AD (Table 2).

**Table 2:** Known AD risk genes, core mouse phenotypes, and microbial metabolites that were used to evaluate the context-sensitive network-based prioritization algorithm

Evaluation dataset 1 (AD risk genes)	Evaluation dataset 2 (AD phenotypes in mouse)	Evaluation dataset 3 (AD microbial metabolites)
A2M, ABCA7, ACE, ADAM10, APBB2, APOE, APP, BLMH, HFE, MPO, MT-ND1, NOS3, PAXIP1, PLAU, PLD3, PRNP, PSEN1, PSEN2, SORL1, TF, TNF, VEGFA	Amyloid beta deposits, amyloidosis Cerebral amyloid angiopathy Neurofibrillary tangles Tau protein deposits Neurodegeneration Neuron degeneration Gliosis Astrocytosis Microgliosis Abnormal synaptic transmission Abnormal long-term potentiation Abnormal long-term depression	3,4-Dihydroxybenzeneacetic acid 3-Hydroxybutyric acid Acetylcholine d-Glutamic acid Dopamine Folic acid Gamma-aminobutyric acid l-Lactic acid Mannitol Putrescine Pyruvic acid Succinic acid

Microbial metabolite-associated genes were then obtained from the STITCH (Search Tool for Interactions of Chemicals) database by mapping microbial metabolites from HMDB to chemicals in STITCH. The STITCH database contains 15,473,939 chemical–gene associations found in the human body, representing 473,602 chemicals and 18,701 human genes [31] (data accessed in October 2020). Among the 220 microbial human metabolites, 216 were mapped to chemicals in STITCH. For example, three SCFAs (acetic acid, butyric acid, propionic acid) are associated with 3926, 815, and 459 human genes, respectively. Bile acids (deoxycholic acid, taurodeoxycholic acid, chenodeoxycholic acid) are associated with 258, 57, and 129 human genes, respectively. MGN is a weighted network consisting of 12,130 nodes (216 metabolite nodes, 11,914 gene nodes) and 67,012 edges (metabolite–gene associations), with edge weights directly derived from the chemical–gene association weights (ranging from 100 to 999).

##### Mutational phenotype**–**gene network (PhenGN)

PhenGN was constructed from MGD that contains The Mouse Genome Database (MGD) database large amounts of phenotypic descriptions of systematic genetic knockouts in mouse models [32]. A total of 517,381mutational/causal phenotype**–**gene annotations (9982 phenotypes and 11,021 mapped human genes) were obtained from MGD. We have used these strong causal gene**–**phenotype associations for screening and validating functional effects of drugs and microbial metabolites on disease phenotypes [33–36]. In this study, we developed a network-based model to model and assess phenotypic effects of the interactions of microglia, microbial metabolites, human genes, and pathways on AD-specific phenotypes*.* PhenGN allows us to interrogate causal microglia**–**gut**–**brain connections and examine how these interactions affect specific AD phenotypes such as “amyloid beta deposits**,**” “tau protein deposits**,**” or “neurodegeneration**.**” PhenGN is unweighted (same weights for all phenotype**–**gene connections on the network) and consisted of 21,003 nodes (9982 phenotype nodes, 11,021 gene nodes) and 517,381 edges.

##### Pathway–gene network (PathGN)

We obtained gene**–**pathway associations from the Molecular Signatures Database (MSigDB) (data accessed in June 2020). MSigDB is a comprehensive resource of annotated pathways and gene sets [37]. PathGN is unweighted (same weights for all pathway**–**gene connections on the network) and consisted of 10,197 nodes (8868 gene nodes, 1329 pathway nodes) and 66,293 edges (gene**–**pathway associations).

##### Protein**–**Protein Interaction Network (PPIN)

PPIN was directly constructed from the protein**–**protein interactions from the human protein reference database [38]. PPIN is weighted and consisted of 22,982 gene nodes and 382,256 gene**–**gene edges, with weights (ranging from 100 to 999) directly obtained from BioGrid.

##### Links between MGN, PhenGN, PathGN, and PPIN

These sub-networks were connected through common gene nodes. For example, MGN are linked to the PhenGN network through shared gene nodes.

#### Network prioritization and evaluation

##### Network prioritization

The goal of this study is to identify microbial metabolites associated with abnormal microglia function and physiology that affect AD at the genetic, functional, and phenotypic levels. For a given input related to disrupted microglia physiology and function (i.e., *microgliosis*, *abnormal microglial cell physiology*, *abnormal microglial cell morphology*), the algorithm prioritized genes, phenotypes, and microbial metabolites from MGPPN based on the context-sensitive network-based ranking algorithm that we previously developed [39–43]. The random walk-based approach is briefly described below. More details are in our previous papers [23, 24, 30, 31, 44]. The movements of a random walker between any two sub-networks were regulated with jumping probabilities ${\lambda}_{N_i{N}_j}$ (*N*_*i*_ and *N*_*j*_ can be any of the four sub-networks). For example, if a random walker stands on a gene node on *MGN*, which is connected with both *PhenGN*, *PathGN*, and *PPIN*, it has the option to walk to *PhenGN* with the probability *λ*_12_, to *PathGN* with the probability *λ*_13_, and to *PPIN* with the probability *λ*_14_ or stay within *MGN* with the probability 1 − *λ*_12_ − *λ*_13_ − *λ*_14_. Given the seed node(s)/inputs, the ranking score for each node is iteratively updated by:

1 $${S}_{k+1}=\alpha {M}^T{S}_k+\left(1-\alpha \right){S}_0,$$

*S*_*k* + 1_ is the score vector at step *k* + 1, *S*_0_ is the initial vector, and 1 − *α* is the restarting probability, and *M* is the transition matrix. The transition matrix *M* was calculated as follows in Eq. 2 and 3):

2 $$M=\left[\begin{array}{ccc}{M}_1& {M}_{1.}& {M}_{1n}\\ {}{M}_{m1}& {M}_m& {M}_{mn}\\ {}{M}_{n1}& {M}_{n.}& {M}_n\end{array}\right]$$

*M* consisted of 16 sub-matrices, each contains the transition probabilities within or between four sub-networks. Each sub-matrix was calculated by normalizing the rows in the adjacency matrix of the corresponding sub-networks using the jumping probabilities. Specifically, the off-diagonal sub-matrices corresponded to the bipartite networks that connected each two networks. These sub-matrices were calculated by first normalizing the rows of the bipartite network ${A}_{N_i{N}_j}$ and then weighing each row by the jumping probability ${\lambda}_{N_i{N}_j}$:

3 $${\left({M}_{N_i{N}_j}\right)}_{kl}=\left\{\begin{array}{c}{\lambda}_{N_i{N}_j}{\left({A}_{N_i{N}_j}\right)}_{kl}/{\sum}_l{\left({A}_{N_i{N}_j}\right)}_{kl}\kern1.25em {\sum}_l{\left({A}_{N_i{N}_j}\right)}_{kl}\ne 0\\ {}0\kern11.5em otherwise\end{array}\right.$$

The diagonal sub-matrices corresponded to the transition probabilities within each one of the four sub-networks and were calculated by first normalizing the rows of adjacency matrix for MGN, PhenGN, PathGN, and PPIN and then weighing the rows by the probability of staying in the same network:

4 $${\left({M}_{N_i}\right)}_{kl}=\left(1-\sum {I}_{N_j}{\lambda}_{N_i{N}_j}\right){\left({A}_{N_i}\right)}_{kl}/{\sum}_l{\left({A}_{N_i}\right)}_{kl}$$

In Eq. 4, ${A}_{N_i}$ is the adjacency matrix of the sub-matrix *N*_*i*_, and ${I}_{N_j}$is an indicator function, whose value is 1 if the *k*th row of ${A}_{N_i{N}_j}$contains at least one non-zero element. The output from the context-sensitive network-based algorithm was a list of microbial metabolites, genes, and phenotypes prioritized based on their genetic, functional, phenotypic, and microbial relevance to disrupted microglia function based on the inputs: “microgliosis,” “abnormal microglial cell physiology,” and “abnormal microglial cell morphology.”

##### Evaluation

It is unknown which microbial metabolites are associated with microglia–brain interactions in AD. However, it is known that microglial function is closely associated with AD etiology. We evaluated the context-sensitive network-based ranking algorithm by showing that abnormal microglia function and physiology are indeed significantly involved in AD pathology at both genetic and phenotypic levels. We also showed that microbial metabolites are involved in AD through microglia functions by demonstrating the known AD-associated microbial metabolites were ranked highly by the algorithm for the inputs (“microgliosis,” “abnormal microglial cell physiology,” and “abnormal microglial cell morphology”).

**Evaluate microglia–AD interactions at the phenotypic level**

There is a large, ongoing effort to characterize AD models and identify core AD-related phenotypes. We obtained all core phenotypes from commonly used AD mouse models [45]. These phenotypes include a range of core AD-related phenotypes including plaques, tangles, neuronal loss, gliosis, synaptic loss, changes in LTP/LTD, and cognitive impairment (Table 2). We evaluated whether the prioritization algorithm ranked these AD phenotypes highly among a total of 9982 phenotypes on the network, when three microglia-related phenotypes (“abnormal microglial cell morphology,” “abnormal microglial cell physiology,” and “microgliosis”) were used as inputs separately and combined. Mean ranking and median rankings of these 13 phenotypes among the list of prioritized 9982 phenotypes were calculated and compared to the average ranking of 50% expected from random ranking. Significance was calculated using the two-sample *t*-test.

**Evaluate microglia–AD interactions at the genetic level**

Hundreds of genes are known to be associated with AD. In this study, we used genes known to be strongly associated with AD to evaluate microglia–AD interactions at the genetic level. We obtained all AD-associated genes (22 in total) from two well-established disease genetics databases *Online Mendelian Inheritance in Man* (OMIM) [46] and ClinVar [47] (Table 2). OMIM contains all known mendelian disorders and over 16,000 genes. ClinVar contains disease–gene associations with supporting evidence of clinical significance. While this list may not be complete, we used it to evaluate whether the prioritization algorithm ranked these AD genes highly among a total of 22,982 genes, when three microglia-related phenotypes (“abnormal microglial cell morphology,” “abnormal microglial cell physiology,” and “microgliosis”) were used as inputs separately and combined. Mean ranking and median rankings of these 22 AD genes among the list of prioritized 22,982 genes were calculated and compared to the average ranking of 50% expected from random ranking. Significance was calculated using the two-sample *t*-test.

**Evaluate microglia–AD interactions at the microbial metabolism level**

We obtained 12 AD–microbial metabolite associations from HMDB [30], which were manually curated from published biomedical research articles (Table 2). Note that these AD-associated microbial metabolites were produced by microbes, but not necessarily exclusively so. We evaluated whether the prioritization algorithm ranked these AD-associated microbial metabolites highly among a total of 216 microbial metabolites on the network, when three microglia-related phenotypes (“abnormal microglial cell morphology,” “abnormal microglial cell physiology,” and “microgliosis”) were used as inputs separately and combined. Mean ranking and median rankings of these 12 AD-associated microbial metabolites among the list of prioritized 216 microbial metabolites were calculated and compared to the average ranking of 50% expected from random ranking. Significance was calculated using the two-sample *t*-test.

#### Identify microglia–microbial metabolite–gene–pathway–phenotype interactions in AD

We identified microbial metabolite–microglia–gene–pathway–phenotype interactions in AD based on top-ranked microbial metabolites (top 20% in this study). For each top-ranked microglia-associated microbial metabolite (e.g., acetic acid), we obtained its associated genes from the STITCH (Search Tool for Interactions of Chemicals) database [31]. The confidence score of chemical–gene associations in STITCH ranges from 100 to 999. In this study, we used the cutoff score of 500. Since a microbial metabolite can target many genes and pathways, many of which are not involved in AD, we then filtered microbial metabolite-associated genes using the 22 known AD risk genes (Table 2) in order to find AD genes targeted by the microbial metabolite. For example, at the cutoff value of 500, butyric acid is associated with 1787 genes, among which three are strong AD genes (APP, NOS3, VEGFA). From microbial metabolite-associated AD genes, microbial metabolite–gene–pathway–phenotype associations were identified by linking microbial metabolite-associated AD genes to their pathways based on gene–pathway associations from Molecular Signatures Database (MSigDB) [37] and gene–phenotype annotations from the Mouse Genome Database (MGD) database [32]. For example, based on the acetic acid–APP association, we obtained “acetic acid-APP-inflammasomes-amyloid beta deposits” and “acetic acid-APP-GPCR ligand binding-amyloid beta deposits” interactions that are mediated by microglia.

#### Case study of SCFAs

Short-chain fatty acids (SCFAs) are the end products of fermentation of dietary fibers by gut microbiota and play a major role in the maintenance of gut and immune homeostasis [23, 24]. Both in vitro and in vivo studies suggested that SCFA may play a key role in microbiota–gut–brain crosstalk [20, 25]; however, the mechanisms through which SCFAs might influence brain functioning in AD have not been fully elucidated [44]. We first examined whether three SCFAs (acetic acid, butyric acid, and propionic acid) were ranked highly by the network-based ranking algorithm given the input microglia-associated phenotypes. We then examined top AD-associated genes, pathways, and phenotypes targeted by each of these three SCFAs.

## Results

### Validation: disrupted microglia function and physiology are significantly associated with AD at both genetic and phenotypic levels

As a validation step, we examined how abnormal microglial function and physiology were associated with AD-specific pathological phenotypes and causal genes. Using three seeds (“microgliosis,” “abnormal microglial cell physiology,” “abnormal microglial cell morphology”) alone or combined, the network-based ranking algorithm prioritized a total of 10,122 mouse mutational phenotypes. Among the prioritized phenotypes, the 13 AD-associated phenotypes (described in the “Methods” section) ranked significantly highly than random expectation (Table 3). For example, when the seed “microgliosis” was used as the input, the AD-associated phenotype “neurodegeneration,” “amyloid beta deposits,” and “tau protein deposits” ranked at the top 0.30%, 2.36%, and 10.42%, respectively. These findings are consistent with published findings that microglia are involved in AD pathologies, including tau protein spreading [48]. These results confirmed the validity of our network-based ranking algorithms in studying etiologies of AD and that microglia dysfunction is mechanistically involved in AD pathologies (“microglia-AD axis”).

**Table 3:** Mean and median rankings of 13 AD-associated mouse phenotypes among a total of 10,122 prioritized mouse mutational phenotypes for input phenotype “abnormal microglial cell morphology,” “abnormal microglial cell physiology,” and “microgliosis” separately or combined. A mean value of top 6.52% means that on average the 13 AD phenotypes ranked in the top 6.52% among the prioritized list of 9982 mouse phenotypes, which was significantly higher than the expected ranking of 50% based on random ranking

Input	Mean (top %)	Median (top %)	***P***-value
Abnormal microglial cell physiology	6.52	2.27	2.67E−10
Abnormal microglial cell morphology	7.68	3.17	5.58E−09
Microgliosis	6.38	2.31	1.04E−09
Combined	6.36	2.32	3.72E−09

We then examined how abnormal microglia function and physiology was associated with AD-specific causal genes. Using three seeds (“microgliosis,” “abnormal microglial cell physiology,” “abnormal microglial cell morphology”) alone or combined, the network-based ranking algorithm prioritized a total of 23,995 human genes. The 22 AD-associated genes (Table 2) ranked significantly highly among the list of prioritized human genes (Table 4). For example, when the seed “microgliosis” was used as the input, AD-associated genes “APP,” “PSEN1,” “PRNP,” “PSEN2,” “APOE,” “TNF,” and “VEGFA” ranked at the top 0.004%, 0.02%, 0.04%, 0.10%, 0.38%, 0.39%, and 0.59% among a total of 23,995 prioritized genes, respectively. The fact that many AD-associated genes ranked highly for all inputs indicates that abnormal microglial function is involved in multiple AD-related genetic functions. However, not all AD-associated genes ranked highly. For example, when the seed “microgliosis” was used as the input, gene MT-ND1, the NADH dehydrogenase subunit 1 gene, ranked at the top 60.23%, suggesting that microgliosis may contribute to AD etiology independent of oxidative phosphorylation in mitochondria.

**Table 4:** Rankings of the 22 AD-associated genes among the list of 23,995 prioritized genes when the input phenotypes “abnormal microglial cell morphology,” “abnormal microglial cell physiology,” and “microgliosis” were used separately or combined. A mean value of top 10.59% means that on average the 22 AD genes ranked in the top 10.59% among the prioritized list of 22,982 genes, which was significantly higher than the expected ranking of 50% based on random ranking

Input	Mean (top %)	Median (top %)	***P***-value
Abnormal microglial cell physiology	10.59	5.31	3.41E−11
Abnormal microglial cell morphology	11.30	7.30	1.92E−11
Microgliosis	113.1	9.48	3.39E−10
Combined	11.49	7.81	6.47E−11

### Microbial metabolites underlie microglia–brain interactions in AD

The 12 known AD-associated microbial metabolites (described in the “Methods” section) ranked significantly highly among a total of 220 prioritized microbial metabolites for three inputs (“abnormal microglial cell morphology,” “abnormal microglial cell physiology,” and “microgliosis”) separately or combined (Table 5). These results supported our hypothesis that microbial metabolites are involved in microglia-mediated AD pathologies.

**Table 5:** Evaluation of the prioritization algorithm using the 12 known AD-associated microbial metabolites among 220 microbial metabolites. A mean value of top 15.08% means that on average the 12 AD-associated metabolites ranked in the top 15.08% among the prioritized list of 216 microbial metabolites, which was significantly higher than the expected ranking of 50% based on random ranking

Input	Mean (top %)	Median (top %)	***P***-value
Abnormal microglial cell physiology	15.08	13.89	3.19E−07
Abnormal microglial cell morphology	15.39	11.11	1.84E−06
Microgliosis	15.63	11.11	4.16E−06
Combined	14.39	11.34	1.06E−06

Among the 220 microbial metabolites present in human analyzed, the top 44 microbial metabolites for the combined input (“microgliosis,” “abnormal microglial cell physiology,” “abnormal microglial cell morphology”) are listed in Table 6. The entire list of prioritized microbial metabolites for the combined input “microgliosis,” “abnormal microglial cell physiology,” and “abnormal microglial cell morphology” is in Supplement_S1. Among the top-ranked microbial metabolites that were predicted to be associated with microglia–AD interactions include three short-chain fatty acids or SCFAs (acetic acid, propionic acid, butyric acid) that are metabolic products of bacterial dietary fiber fermentation. Many others are produced by both microbes and human hosts, such as dopamine and gamma-aminobutyric acid. Our results are consistent with the recently published finding of protective roles of SCFAs in AD-type beta-amyloid neuropathological mechanisms [16].

**Table 6:** Top 44 (top 20%) predicted microbial metabolites for the combined input “microgliosis,” “abnormal microglial cell physiology,” and “abnormal microglial cell morphology”

R	Metabolite	R	Metabolite	R	Metabolite	R	Metabolite
1	Acetic acid	12	Ascorbic acid	23	Butyric acid	34	Sulfamethoxazole n1-glucuronide
2	Ethanol	13	Mannitol	24	Isopropyl alcohol	35	Salicyluric acid
3	d-Alanine	14	Epinephrine	25	Serotonin	36	Propionic acid
4	d-Glutamic acid	15	Methane	26	Folic acid	37	d-Proline
5	Hydrogen	16	Norepinephrine	37	Phenylethylamine	38	3-Hydroxydodecanoic acid
6	Dopamine	17	Gamma-aminobutyric acid	28	2-Phenylethanaminium	39	1-Butanol
7	d-Lactic acid	18	4-Hydroxybutyric acid	29	Phenol	40	3-Hydroxybutyric acid
8	l-Lactic acid	19	Oxoglutaric acid	30	Chenodeoxycholic acid 3-sulfate	41	Zeaxanthin
9	Hydroxypropionic acid	20	Glutaric acid	31	3,4-Dihydroxybenzeneacetic acid	42	Cis,cis-muconic acid
10	Acetaldehyde	21	Melatonin	32	Succinic acid	43	Trans-trans-muconic acid
11	Histamine	22	Trehalose	33	Muramic acid	44	Nndole

### Microglia–microbial metabolite–gene–pathway–phenotype interactions in AD

A total of 8094 microglia–microbial metabolite–gene–pathway–phenotype interactions in AD were identified for the top 20% (top 44) ranked microbial metabolites (Supplement_S2). For example, acetic acid, the top microglia-associated microbial metabolite, is involved in 446 microglia–microbial metabolite–gene–pathway–phenotype interactions in AD, including *microglia-acetic acid-APP-inflammasomes-tau protein deposits*, *microglia-acetic acid-NOS3-calcium signaling-abnormal long-term potentiation*, *microglia-acetic acid-VEGFA-cytokine-cytokine interaction-astrocytosis*, among others. The top 10 most frequently targeted AD genes, pathways, and phenotypes by top 44 microbial metabolites are shown in Table 7. For example, APP and TNF are targeted by 21 and 9 of the top 44 microbial metabolites, respectively. Pathways “Alzheimer’s disease” and “Amyloids” are targeted by 22 and 21 of the top 44 microbial metabolites, respectively. Phenotypes “abnormal synaptic transmission” and “amyloid beta deposits” are targeted by 27 and 22 of the top 44 microbial metabolites, respectively.

**Table 7:** Top 10 most frequently targeted AD genes, pathways, and mutational phenotypes by top-ranked (top 20% or top 44) microglia-associated microbial metabolites. The numbers of microbial metabolites were shown in the parenthesis

Top 10 AD genes associated with microbial metabolites	Top 10 AD pathways associated with microbial metabolites	Top 10 AD phenotypes associated with microbial metabolites
APP (21), NOS (16), VEGFA (12), TNF (9), TF (8), MPO (6), PLAU (3), APOE (2), ACE (2), BLMH (2)	Hemostasis (28), Glypican 1 network (24), response to elevated platelet cytosolic Ca2+ (24), platelet activation, signaling and aggregation (24), Alzheimer’s disease (22), amyloids (21), deregulation of CDK5 in Alzheimer’s disease (21), innate immune system (21), VEGF signaling pathway (21), inflammasomes (21)	Abnormal synaptic transmission (27), abnormal long-term potentiation (27), gliosis (24), astrocytosis (24), amyloidosis (22), amyloid beta deposits (22), microgliosis (21), tau protein deposits (21), neuron degeneration (13), cerebral amyloid angiopathy (2)

### Short-chain fatty acids (SCFAs)

Three SCFAs ranked in the top 44 microglia-associated microbial metabolites: acetic acid (top 1), butyric acid (top 23), and propionic acid (top 36) (Table 6). APP gene is targeted by three SCFAs; a total of 8 (out of 12) AD phenotypes (*abnormal long-term potentiation*, *abnormal synaptic transmission*, *amyloid beta deposits*, *amyloidosis*, *astrocytosis*, *gliosis*, *microgliosis*, and *tau protein deposits*) are associated with three SCFAs. The 30 shared pathways targeted by three SCFAs (Table 8) include known AD-related genetic pathways, including “Alzheimer’s disease,” amyloids, and immune systems. These results indicated that SCFAs are mechanistically involved in microglia-mediated gut–microbiota–brain interactions in AD at both genetic, functional, and phenotypic levels.

**Table 8:** Genetic pathways targeted by three SCFAs

Alzheimer’s disease	GPCR ligand binding	Platelet activation, signaling, and aggregation
Amyloids	Hemostasis	Platelet amyloid precursor protein pathway
Caspase cascade in apoptosis	Immune system	Response to elevated platelet cytosolic Ca2+
Deregulation of CDK5 in Alzheimer’s disease	Inflammasomes	Signaling by GPCR
G alpha (i) signaling events	Innate immune system	TAK1 activates NFkB by phosphorylation and activation of IKKs complex
G alpha (q) signaling events	NFkB and MAP kinases activation mediated by TLR4 signaling repertoire	TLR4 signaling
Gastrin-CREB signaling pathway via PKC and MAPK	NLRP3 inflammasome	Toll-like receptor cascades
Glycosylation end product receptor signaling	Nucleotide-binding domain, leucine-rich repeat containing receptor (NLR) signaling pathways	TRAF6 mediated induction of NFkB and MAP kinases upon TLR7/8 or 9 activation
Glypican 1 network	p75(NTR)-mediated signaling	TRAF6 mediated NF-kB activation
GPCR downstream signaling	Peptide ligand-binding receptors	TRIF mediated TLR3 signaling

## Discussion

We developed a knowledge-driven context-sensitive network-based framework to identify microglia–microbial metabolite–gene–pathway–phenotype interactions in AD. The approach innovatively repurposed publicly available datasets collected for other purposes to study the gut–microbiota–microglia–brain interactions in AD. We validated this computational framework by showing that abnormal microglia function/physiology are indeed significantly associated with AD at both the genetic, phenotypic, and microbial metabolism levels. We identified a total of 8094 potential microglia–microbial metabolite–gene–pathway–phenotype interactions in AD for the top-ranked microbial metabolites.

There is evidence in the literature associating many of the top-ranked 44 microglia-associated microbial metabolites with AD. For example, in vitro administration of SCFAs (microbial fermentation metabolites) regulated microglia homeostasis and obstructed Aβ protein aggregation [25]. Supplementation of SCFAs in germ-free mice rescued the immature genetic and morphological phenotype of microglia [20]. Three SCFAs may play a key role in microbiota–gut–brain crosstalk [44] and have protective roles in AD-type beta-amyloid neuropathological mechanisms [48]. Despite such growing links, the mechanisms underlying how gut microbial metabolites interact with microglia and host genetics in promoting or protecting against AD remain largely unknown. Our analysis provided potential mechanistic insights into how microbial metabolites SCFAs (acetic acid, butyric acid, and propionic acid) are involved in microglia functions in AD at genetic, functional, and phenotypic levels: they are among top microbial metabolites prioritized for microglia functions, all target APP gene and AD-related pathways including “Alzheimer’s disease,” “Amyloids,” and “Immune systems” and are associated with multiple AD-related phenotypes including “abnormal long term potentiation,” “abnormal synaptic transmission,” “amyloid beta deposits,” “amyloidosis,” “astrocytosis,” “gliosis,” “microgliosis,” and “tau protein deposits.” Details of these links are provided in Supplement_S2.

Many studies reported associations among alcohol consumption, dementia, and cognitive decline [49–51], but the findings remain controversial [52]. The effect of alcohol on cognitive functions and AD pathogenesis and the underlying molecular mechanisms remain elusive. In our study, ethanol ranked at top 2 among microglia-associated microbial metabolites. It targets NOS3 and VEGFA and is associated with multiple AD core phenotypes including abnormal long-term potentiation, abnormal synaptic transmission, astrocytosis, gliosis, and neuron degeneration (see Supplement_S2). It remains an open question as to in what degree ethanol produced by microbiota play a role in AD pathogenesis and whether previous controversial findings of alcohol–AD associations could be partially explained by different levels of microbial ethanol production among different individuals.

The roles of glutamic acid in AD remain unclear. Our analysis suggests that glutamic acid targets APP and may be associated with amyloidosis, amyloid beta deposits, tau protein deposits, and neurodegeneration. The dopaminergic system and dopaminergic deficit may be involved in cognitive decline in AD [53, 54]. Concentrations of several monoaminergic neurotransmitters, their precursors, and metabolites such as dopamine and serotonin were significantly reduced in AD versus control patients [55]. Dopamine and serotonin were ranked at top 6 and 25 among 220 prioritized microglial-associated microbial metabolites. However, it remains unclear how gut microbiota-produced dopamine and serotonin, which normally are unable to cross the blood–brain barrier, exert their effects on the brain by restoring deficits of human dopaminergic transmitter systems.

A higher plasma level of lactic acid was found in significantly higher levels in Down syndrome patients with AD than Down syndrome patients without AD, suggesting that lactic acid may be involved in AD [56]. Our results showed that lactic acid targets APP and AD-associated pathways and are associated with amyloidosis, amyloid beta deposits, tau protein deposits, neurodegeneration, and other core AD phenotypes. These results are consistent with a prior study showing that increased levels of lactic acid lead to amyloidogenesis in AD by inducing APP aggregates [57].

### Limitations

Our study has several limitations that warrant further discussion. First, our study is purely “in silico” and generated a large number of possible hypotheses (8094 potential microglia–microbial metabolite–gene–pathway–phenotype interactions in AD). We robustly evaluated the knowledge-driven network-based ranking algorithm by showing that microglia are involved in AD at both genetic and phenotypic levels and that microbial metabolites are involved in AD through microglial function. However, we could not directly evaluate the identified microglia–microbial metabolite–gene–pathway–phenotype interactions in AD since we currently have limited knowledge of how gut microbial metabolites contribute to AD through microglial functions at genetic, functional, and phenotype levels. Future studies on the role of top-ranked microglia-related genes and microbial metabolites in an experimental rodent model of AD may provide a better understanding of the impact of microglia–microbial metabolite–gene–pathway–phenotype interactions in the pathogenesis of AD.

Second, our findings are largely associational. Although the networks incorporated strong causal/mutational gene–phenotype relationships from MGD, other relationships on the network including chemical–gene, gene–gene, and gene–pathway are associational. In addition, network-based ranking algorithms that prioritized entities on the network for a given input are based on associational analysis. To translate these findings into AD diagnosis, prevention, and treatment, it is necessary to establish cause–effect relationships of the identified microglia–microbial metabolite–gene–pathway–phenotype interactions in experimental models of AD and identify specific gut bacteria that produce the metabolites. Furthermore, the identified top-ranked AD–microbial metabolites and their associated pathways are not necessarily specific for AD. For example, SCFAs and associated immune functions and pathways are also involved in other diseases including cancers [58].

Third, HMDB is currently the most comprehensive human metabolome database. However, it is not specific for microbial metabolism and contains only 220 metabolites that originated from gut microbiota. The field of human microbial metabolism research is expanding rapidly, with an increasing number of microbial metabolites being identified or predicted by computational methods. For example, AGORA (assembly of gut organisms through reconstruction and analysis) is a resource of genome-scale metabolic reconstructions semi-automatically generated for 773 human gut bacteria [59]. The computationally predicted (while not experimentally confirmed) microbial metabolites in AGORA may significantly broaden our current list of 220 microbial metabolites.

Another rich resource for a list of validated microbial metabolites is the 30 million published biomedical literature. Many microbial metabolites already reported in the literature are not included in the list of 220 microbial metabolites from HMDB [60, 61]. We have recently developed natural language processing, text classification, and network-based approaches to automatically extract and prioritize microbial metabolites from 28 million biomedical articles [60, 61]. Currently, we are manually curating top-ranked microbial metabolites extracted from biomedical literature, in order to update the analysis of microglia–microbial metabolite–gene–pathway–phenotype interactions in AD using an updated list of microbial metabolites.

Fourth, this study used 22 AD risk genes obtained from OMIM and ClinVar as one of the evaluation datasets. Both OMIM and ClinVar contain disease–gene associations with known clinical significance. However, this list of AD genes is not complete. For example, studies showed that rare variants of PLCG2, ABI3, and TREM2 implicate microglial-mediated innate immunity in AD and the list of AD genes did not include these three genes. However, this limitation did not affect the prioritization algorithm as the input to the prioritization algorithm was microglia-associated phenotypes and the list of AD genes was used for evaluation purpose.

In this study, we did not explicitly differentiate early-onset from late-onset AD. The inputs to the network-based algorithms to identify AD-associated microbial metabolites were three phenotypes related to abnormal microglial functions. Studies showed that proliferation and activation of microglia around amyloid plaques is a canonical feature of AD, including both early-onset and late-onset AD [62–64]. While findings from our study suggest that microbial metabolites are involved in microglia-mediated AD pathophysiology in both early- and late-onset AD, future experimental studies are warranted to further tease out how and which microbial metabolites contribute to different aspects of AD.

Fifth, we used 517,381 mouse mutational phenotype–gene annotations from the MGD to construct the PhenGN network and to infer effects of microbial metabolites on AD-related phenotypes. The advantages of MGD data are that these gene–phenotype associations are strong and often causal and that they contain a large number of genes and phenotypes (11,021 human gene homologs, 9982 phenotypes). However, it is known that phenotypes in mouse models do not necessarily resemble the human phenotypes, especially in AD and other neurological disorders. Future work will include incorporating known phenotype–gene associations from human (though limited availability) into the networks to further improve the human relevance of the predicted gut–microbiota–brain–AD interactions.

## Conclusions

We constructed a context-sensitive network that integrates and models our existing knowledge of semantic relationships among tens of thousands of genes, phenotypes, metabolites, and pathways. We prioritized and identified 8094 potential microglia–microbial metabolite–gene–pathway–phenotype interactions in AD, which provide evidence that multiple gut microbial metabolites are involved in microglia-mediated AD pathophysiology through different genetic and functional mechanisms that are finally converged on several AD-specific phenotypes including “amyloid beta deposits,” “amyloidosis,” “microgliosis,” and “tau protein deposits.” To the best of our knowledge, our study represents the first computational approach to comprehensively characterize the complex gut–microbial metabolite–microglia–gene–pathway–phenotype–brain connections in AD, by innovatively repurposing large amounts of publicly available data collected for other purposes. As our knowledge of genetics and genomics of diseases and chemicals evolve, the network structures that capture our current knowledge of gene–phenotype, chemical–gene, gene–gene, gene–pathway, and metabolite–disease associations will surely also change. Consequently, microglia–microbial metabolite–gene–pathway–phenotype–AD interactions identified based on network prioritization will be further improved. As new data coming in, the context-sensitive network-based approach is highly flexible and dynamic in incorporating new biomedical knowledge into the network. The identification of gut microbial metabolites and the understanding of their role in AD has potential in providing new insights into the basic mechanisms of AD etiology and enable new possibilities for AD prevention and treatment.

## Supplementary Information

**Additional file 1: Supplement S1.** The list of prioritized microbial metabolites for the combined input microgliosis”, “abnormal microglial cell physiology” and “abnormal microglial cell morphology”. **Supplement S2.** A total of 8,094 microglia-microbial metabolite-gene-pathway-phenotype interactions in AD identified for top 20% (top 44) ranked microbial metabolites.

## Data Availability

All the data are provided in the Supplement files.
